# Neutrophils enhance early *Trypanosoma brucei* infection onset

**DOI:** 10.1038/s41598-018-29527-y

**Published:** 2018-07-25

**Authors:** Guy Caljon, Dorien Mabille, Benoît Stijlemans, Carl De Trez, Massimiliano Mazzone, Fabienne Tacchini-Cottier, Marie Malissen, Jo A. Van Ginderachter, Stefan Magez, Patrick De Baetselier, Jan Van Den Abbeele

**Affiliations:** 1 Unit of Veterinary Protozoology, Department of Biomedical Sciences, Institute of Tropical Medicine Antwerp (ITM), Antwerp, Belgium; 2 Laboratory of Microbiology, Parasitology and Hygiene (LMPH), University of Antwerp, Wilrijk, Belgium; 3 Myeloid Cell Immunology Lab, VIB Center for Inflammation Research, Ghent, Belgium; 4 Unit of Cellular and Molecular Immunology, Vrije Universiteit Brussel (VUB), Brussels, Belgium; 5 Ghent University Global Campus, Incheon, South Korea; 6 Laboratory of Tumor Inflammation and Angiogenesis, Center for Cancer Biology, VIB, Leuven, Belgium; 7 Laboratory of Tumor Inflammation and Angiogenesis, Center for Cancer Biology, Department of Oncology, KU Leuven, Leuven, Belgium; 8 Department of Biochemistry, WHO-Immunology Research and Training Center, University of Lausanne, Epalinges, Switzerland; 9 Centre d’Immunologie de Marseille-Luminy, Aix Marseille Université UM2, Inserm U1104, CNRS UMR7280, F-13288 Marseille, France

## Abstract

In this study, *Trypanosoma brucei* was naturally transmitted to mice through the bites of infected *Glossina morsitans* tsetse flies. Neutrophils were recruited rapidly to the bite site, whereas monocytes were attracted more gradually. Expression of inflammatory cytokines (*il1b*, *il6*), *il10* and neutrophil chemokines (*cxcl1*, *cxcl5*) was transiently up-regulated at the site of parasite inoculation. Then, a second influx of neutrophils occurred that coincided with the previously described parasite retention and expansion in the ear dermis. Congenital and experimental neutropenia models, combined with bioluminescent imaging, indicate that neutrophils do not significantly contribute to dermal parasite control and elicit higher systemic parasitemia levels during the infection onset. Engulfment of parasites by neutrophils in the skin was rarely observed and was restricted to parasites with reduced motility/viability, whereas live parasites escaped phagocytosis. To our knowledge, this study represents the first description of a trypanosome infection promoting role of early innate immunological reactions following an infective tsetse fly bite. Our data indicate that the trypanosome is not hindered in its early development and benefits from the host innate responses with the neutrophils being important regulators of the early infection, as already demonstrated for the sand fly transmitted *Leishmania* parasite.

## Introduction

*Trypanosoma brucei* is a protozoan and exclusively extracellular parasite that causes African Trypanosomiasis. Human African Trypanosomiasis or sleeping sickness is a neglected tropical disease restricted to sub-Saharan Africa. It is a vector-borne disease solely transmitted by the blood feeding tsetse fly (*Glossina*). In this fly, *T. brucei* parasites go through a complex developmental cycle in the alimentary tract and salivary glands^1^. Here, the final developmental stage is the non-replicative metacyclic stage that is presumed to be pre-adapted for survival in the mammalian host^2^. Sleeping sickness infection is initiated when these metacyclic parasites are inoculated by the feeding tsetse fly into the skin dermis of the mammalian host^1^. A subpopulation of the inoculated parasites was found to reside in the skin and to proliferate and interact intricately with adipocytes and collagen microfibers and bundles^3^. Both the skin and adipose tissue have very recently been identified as overlooked reservoir tissues for *T. brucei*^3–6^. Parasite migration from the dermal inoculation site resulted in detectable parasite levels in the draining lymph nodes within 18 hours and in the peripheral blood within 42 h in a mouse model^3^. This fast parasite dynamics, starting from the early skin inoculation, confirmed previous work in goats where parasites could be detected in the lymph within 1–2 days after an infective bite^7^.

The skin is a critical barrier between the pathogen and the host and harbors many resident cells and specific immune cells to arrest or limit the infection by secreting inflammatory molecules or by directly killing the pathogen^8^. This includes a set of professional phagocytic cells that are present in the steady state dermis or are recruited following infection. Neutrophils are the most abundant leukocytes in mammalian blood and are prominently and rapidly recruited from the circulation to the site of infection and become effective within hours after infection^9,10^. They have a large armory of anti-pathogen effector pathways that can potentially contribute to parasite control through (i) engulfment and destruction in the phagolysosomes, (ii) secretion of microbicidal factors, (iii) inducing hostile inflammatory conditions and (iv) the release of neutrophil extracellular traps (NETs) whereby the entire nuclear chromatin is expelled into the extracellular environment [Reviewed by^10^]. Neutrophils are also increasingly considered as important regulators of immune function with cell-contact dependent and independent modes of activating/deactivating monocyte-derived cells^11^. During the last decade, the role of the early inflammatory responses and neutrophils in the host skin after a parasite-infected insect vector bite has been extensively studied in *Leishmania*, another trypanosomatid parasite. It was recently discovered that especially bacteria regurgitated by the sand fly trigger an extensive neutrophil recruitment^12^. It was demonstrated that recruited neutrophils efficiently capture *Leishmania* parasites early after the sand fly bite^13^. However, in contrast to being killed, these phagocytosed parasites remained viable and the infected neutrophils efficiently spread the infection^12,13^; neutrophil depletion reduced the ability of the parasite to establish a productive infection. Additionally, recent data showed that different *Leishmania* species elicit distinct neutrophil functions and suggested that neutrophils should be considered as important modulators of the parasite infection outcome (Reviewed by^9,14^).

The early innate immune response in the host skin to the intradermal inoculation of the *T. brucei* parasite and its contribution to parasite control/early development, as well as the trypanosome strategies to overcome this response, are still enigmatic.

Our recent findings have shown that metacyclic *T. brucei* parasites are highly infectious through the intradermal route, in contrast to the blood stream formed parasites that are significantly hampered in establishing an infection following intradermal inoculation^15^. Indeed, this natural intradermal route of trypanosome transmission constitutes a stringent bottleneck for establishment of both *T. brucei* and *T. congolense*, with induced nitric oxide and tumor necrosis factor strongly contributing to the host innate resistance^15^. A number of studies have histologically analyzed the immunological cell composition of *T. brucei* and *T. congolense* induced skin ulcers at the initial parasite inoculation site (also referred to as chancres) that are regularly observed in a range of host species, following an infective tsetse fly bite^16–22^. The formation of this chancre is a phenomenon that is indicated to be mainly driven by the parasite^17,23^.

In this study, we conducted tsetse fly-mediated trypanosome transmissions to the mouse dermis. We observed two different recruitment waves of neutrophils to the dermal site and a more gradual influx of monocytes. Besides documenting neutrophil recruitment, we also functionally addressed the importance of these cells in the infection onset, suggesting a promoting role in the early parasite outgrowth.

## Results

### Neutrophils are recruited in two waves to the dermal site of tsetse-mediated trypanosome inoculation

**Figure 1:**
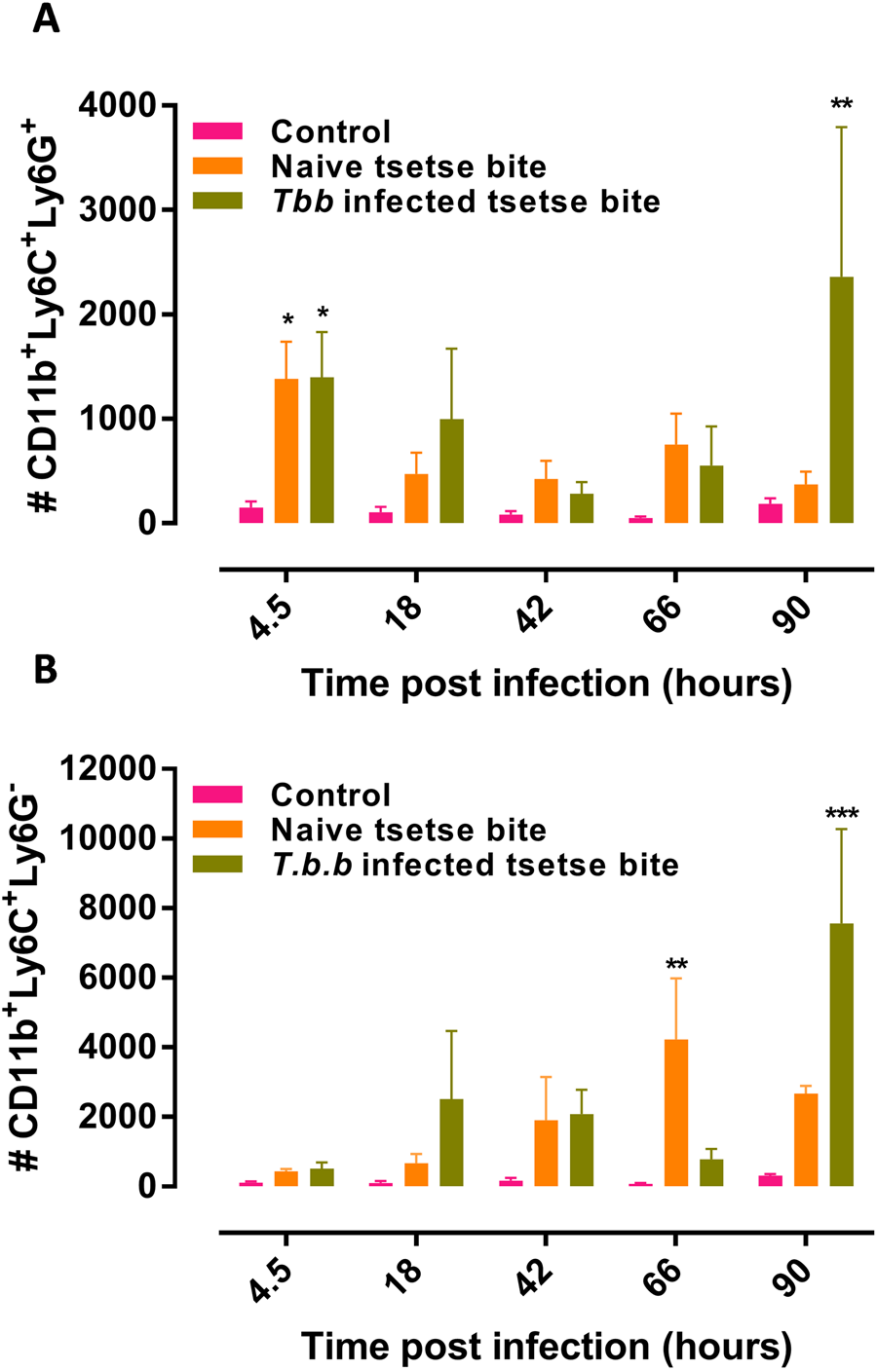
Innate immune cell recruitment to the dermal trypanosome infection site. Numbers of (**A**) neutrophils (CD45^+^CD11b^+^Ly6C^Int^Ly6G^+^) and (**B**) monocytes (CD45^+^CD11b^+^Ly6C^Hi^Ly6G^−^) recovered from ear pinnae of C57Bl/6 mice exposed to the bites of naive or *T.b.b*. SG+ tsetse flies. The gating strategy is shown in Supplemental Fig. S1. Bar charts for each time point are from *n* = 6/group of infected mice and *n* = 3 for the uninfected control mice. The kinetics at the various time points are representative of two independent experiments, the neutrophil recruitment at 4.5 hpi and 90 hpi has been confirmed in respectively 7 and 4 independent experiments. Data in the bar charts are the means ± SEM. Statistical significances indicated above the bars are based on the two-way ANOVA with a Tukey multiple comparison test.

Infections in mice were initiated by the bites of *T.b.brucei* AnTAR1 or AnTat1.1E^dsRed^ infected flies. *T.b.brucei* AnTAR1 infections were the standard infections used for parasitemia follow up whereas the AnTat1.1E^dsRed^ strain allowed us to monitor the parasite kinetics and interactions with immune cell subsets by flow cytometry. Moreover, it was observed previously that parasite densities in the saliva were higher in flies infected with *T.b.brucei* AnTAR1 as compared to *T.b.brucei* AnTat1.1E^dsRed^, yielding respectively a high and low parasite inoculation model^3^. Following exposure of ear pinnae of wild type and LysM-GFP C57Bl/6 mice to infected tsetse fly bites, neutrophils (CD45^+^ CD11b^+^ Ly6C^Int^ Ly6G^+^ LysM-GFP^Hi^ cells) were found to be the main innate immune cell type recruited rapidly [within 4.5 hours post infection (hpi)] to the dermal site of infection (Figs 1A and S1). Exposure of mice to the bites of uninfected tsetse flies gave rise to a similar first neutrophil recruitment (Fig. 1A) suggesting that this response is inherent to the tissue damage as a result of the tsetse bite. In line with the tissue damage-related neutrophil recruitment, natural infection with *T.b.b*. AnTAR1 (high parasite inoculation model) and fluorescently tagged *T.b.b*. AnTat1.1E^dsRed^ (low parasite inoculation model) resulted in comparable neutrophil numbers in the skin (Fig. S2). Monocytes (CD45^+^ CD11b^+^Ly6C^Hi^Ly6G^−^ LysM-GFP^Int^ cells) displayed a more gradual attraction to the dermis (Fig. 1B) with a peak level at 66hpi and 90hpi respectively in the non-infected and infected bite exposed ears. A second strong recruitment of neutrophils at the dermal site was observed at 90 hpi only in mice that were exposed to infected tsetse fly bites, coinciding with the previously described local expansion of a dermal parasite population^3^.

**Figure 2:**
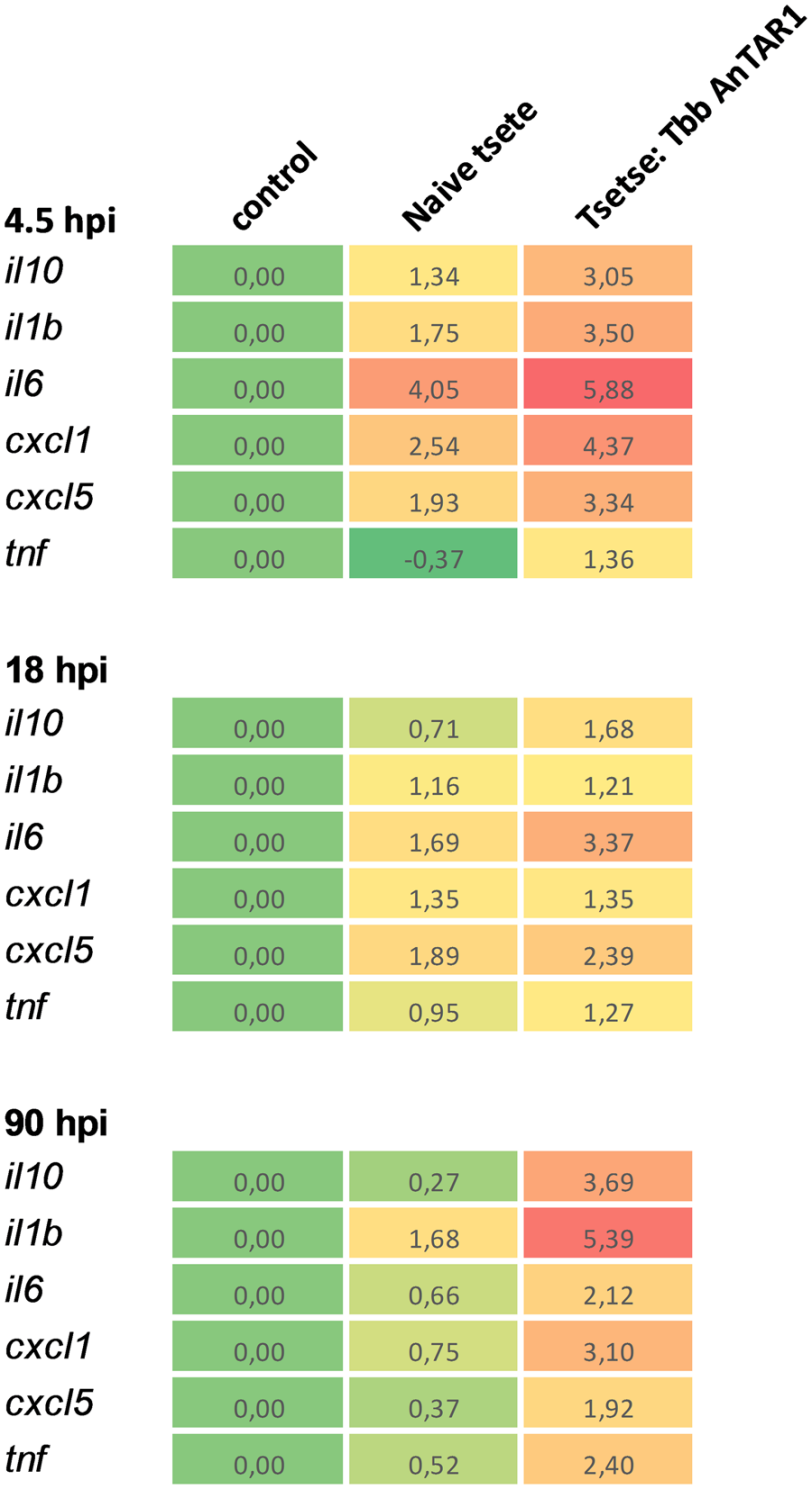
Inflammatory gene transcription at the dermal trypanosome infection site. Heat map representing the expression of differentially regulated inflammatory genes in the dermis of mice exposed to SG^−^ tsetse fly bites or bites of *T.b.b*. AnTAR1 SG^+^ flies at 4.5, 18 and 90 hours post bite exposure. Indicated are the mean log2-fold changes in expression. Individual transcription levels were normalized using the TUBA1A and EEF2 reference genes using the Q base Plus software. Data in the graphs are the means and SEM of 6–12 controls, 6–9 ear tissue samples exposed to naive bites and 6–9 samples exposed to *T.b.b*. AnTAR1.

Quantitative RT-PCR was used to investigate the early inflammatory gene expression at the site of infection in the mammalian host (Fig. 2). Neutrophil recruitment coincided with a locally enhanced transcription of various inflammatory genes, including neutrophil chemokines *cxcl1* and *cxcl5*. Inflammatory cytokines *il1β* and *il6* were transiently and significantly upregulated at the site of parasite inoculation. *Il10* transcription was also found to be rapidly upregulated. These genes were also significantly elevated during the second neutrophil recruitment wave. The above described response was observed by the naive tsetse fly bite as well but was more pronounced upon tsetse fly-mediated inoculation of *T.b.brucei* parasites. Responses in dermis of mice exposed to naive bites were nearly normalized by 90 hpi, in contrast to inflammation in ears harboring a parasite burden.

### Neutrophils do not phagocytose expanding parasites in the early colonization process

**Figure 3:**
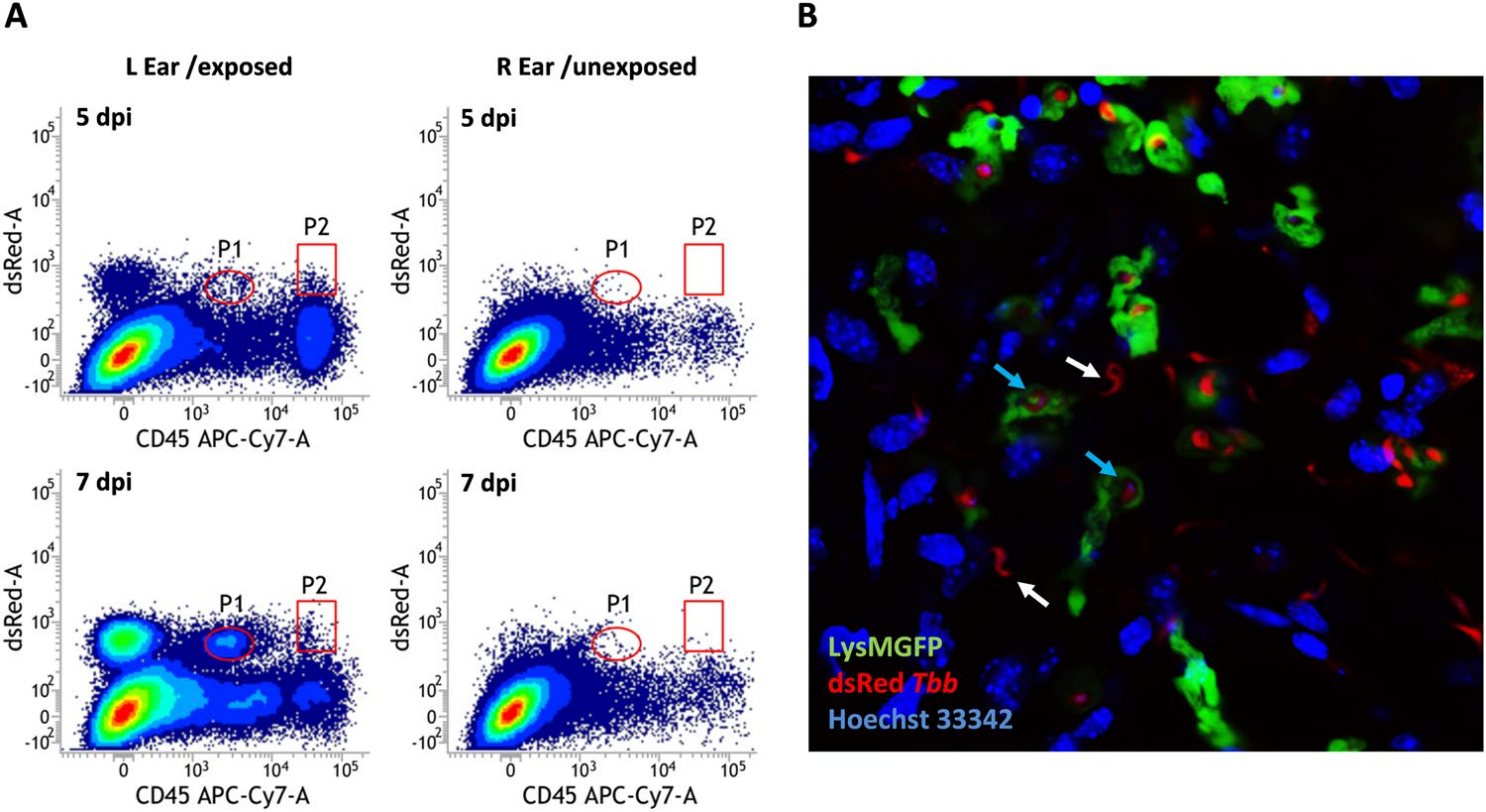
*In vivo* trypanosome phagocytosis in the murine dermis. (**A**) Flow cytometry analysis of *T.b.b*. AnTat1.1E^dsRed^ phagocytosis by white blood cells (CD45^+^/dsRed^+^ cells in the P2 gate) in the dermis of mice infected through the bites of an SG+ tsetse fly. The P1 gate (CD45^dim^/dsRed^+^) represents events with a low FSC/SSC indicating parasitic debris rather than *bona fide* cells. (**B**) Confocal microphotograph of intradermal uptake of *T.b.b*. AnTat1.1E^dsRed^ by LysM-GFP^+^ cells in the infected ear dermis. Uptake occurs if trypanosomes display a reduced motility (see Supplemental Videos S1 and S2). White arrows indicate free living trypanosomes, whereas blue arrows show phagocytosed trypanosomes in the dissected ear tissue.

Neutrophils display a large array of anti-microbial effector functions, including phagocytosis. Parasite uptake by neutrophils at the dermal infection site was evaluated over the early time course of infection. Only marginal levels of phagocytosis were observed, even at very high dermal parasite burdens with a concomitantly high presence of myeloid cells (Fig. 3A). *Ex vivo* confocal imaging inside the ear dermal sheets of *T.b.brucei* AnTat1.1E^dsRed^ infected LysM-GFP mice, harboring GFP-expressing neutrophils and monocytes, confirmed the absence of significant levels of parasite uptake during the early colonization process, despite the patrolling behavior of the GFP^+^ myeloid cells (Video S1). Only upon prolonged *ex vivo* analysis, phagocytosis of trypanosomes by both GFP^Hi^ and GFP^Int^ cells was observed coinciding with reduced parasite motility/viability (Fig. 3B, Video S2). Phagocytosis of naturally transmitted trypanosomes by neutrophils was also evaluated in the blood of mice infected with *T.b.brucei* AnTat1.1E^dsRed^ or *T.b.brucei* AnTat1.1E^TagGFP2^, revealing no significant levels of parasite uptake in the peripheral blood by CD45^+^ cells (Fig. S3). The absence of dsRed or GFP detection in hematopoietic cells could relate to the rapid inactivation of these fluorescent proteins following uptake into a phagolysosome. To exclude this possibility, co-incubation experiments were performed of diluted whole blood with *T.b.brucei* AnTat1.1E^TagGFP2^ parasites that were additionally covalently labeled with the pH-stable dye pHrodo Red (Fig. S4A). Evaluation of uptake in the two fluorescence channels revealed no significant parasite phagocytosis (Fig. S4B).

### Early neutrophil recruitment creates an overall benefit to the onset of a tsetse transmitted trypanosome infection

**Figure 4:**
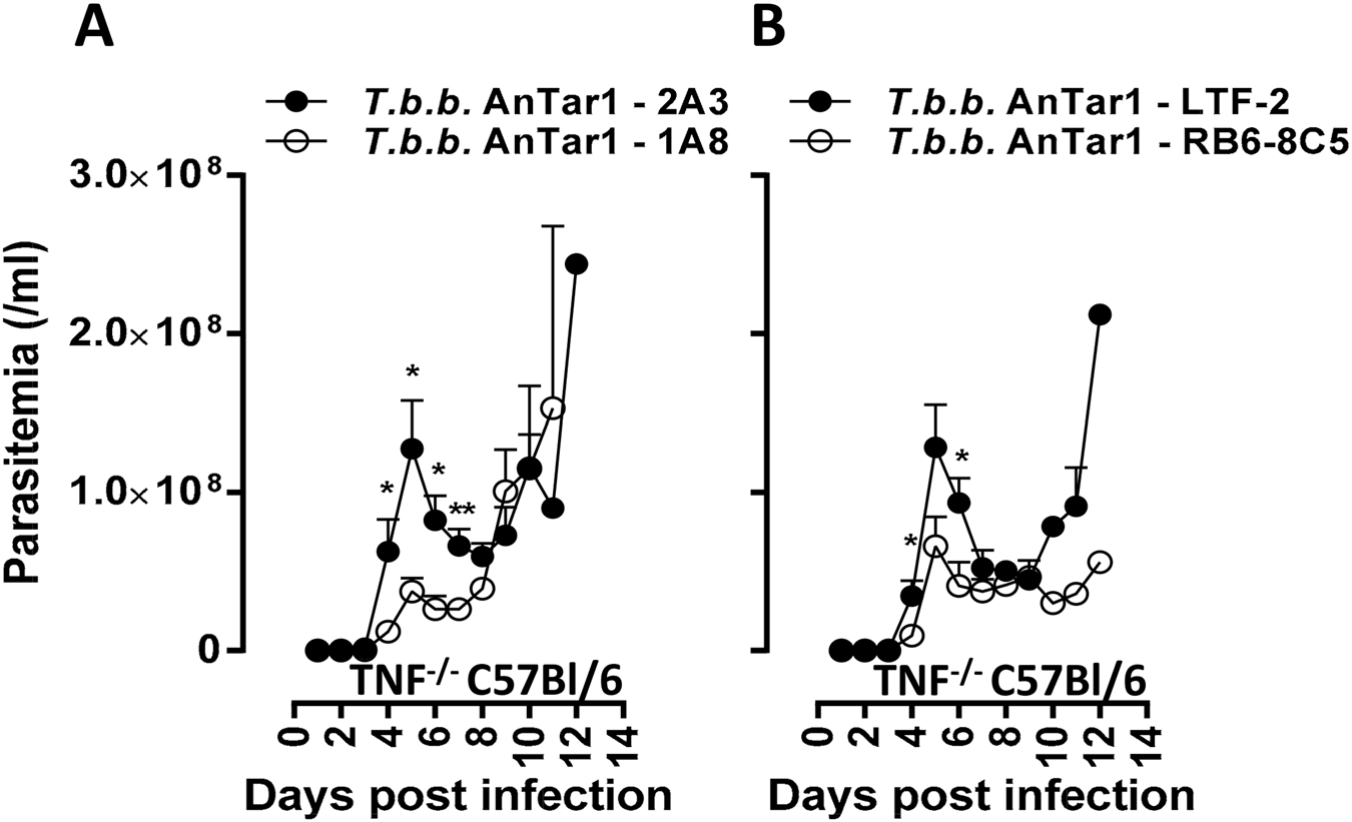
Effect of neutrophil depletion on parasitemia progression in a susceptible mouse model. Parasitemia progression in TNF-α deficient C57Bl/6 mice following a single intraperitoneal injection at day −1 of the depleting antibodies 1A8 (**A**) or RB6-8C5 (**B**) and the appropriate antibody isotype controls (2A3 or LTF-2). Parasitemia data are the means ± SEM of *n* = 6 mice/group. Data are representative of two independent experiments. Statistical significance levels based on the Mann-Whitney test are indicated.

**Figure 5:**
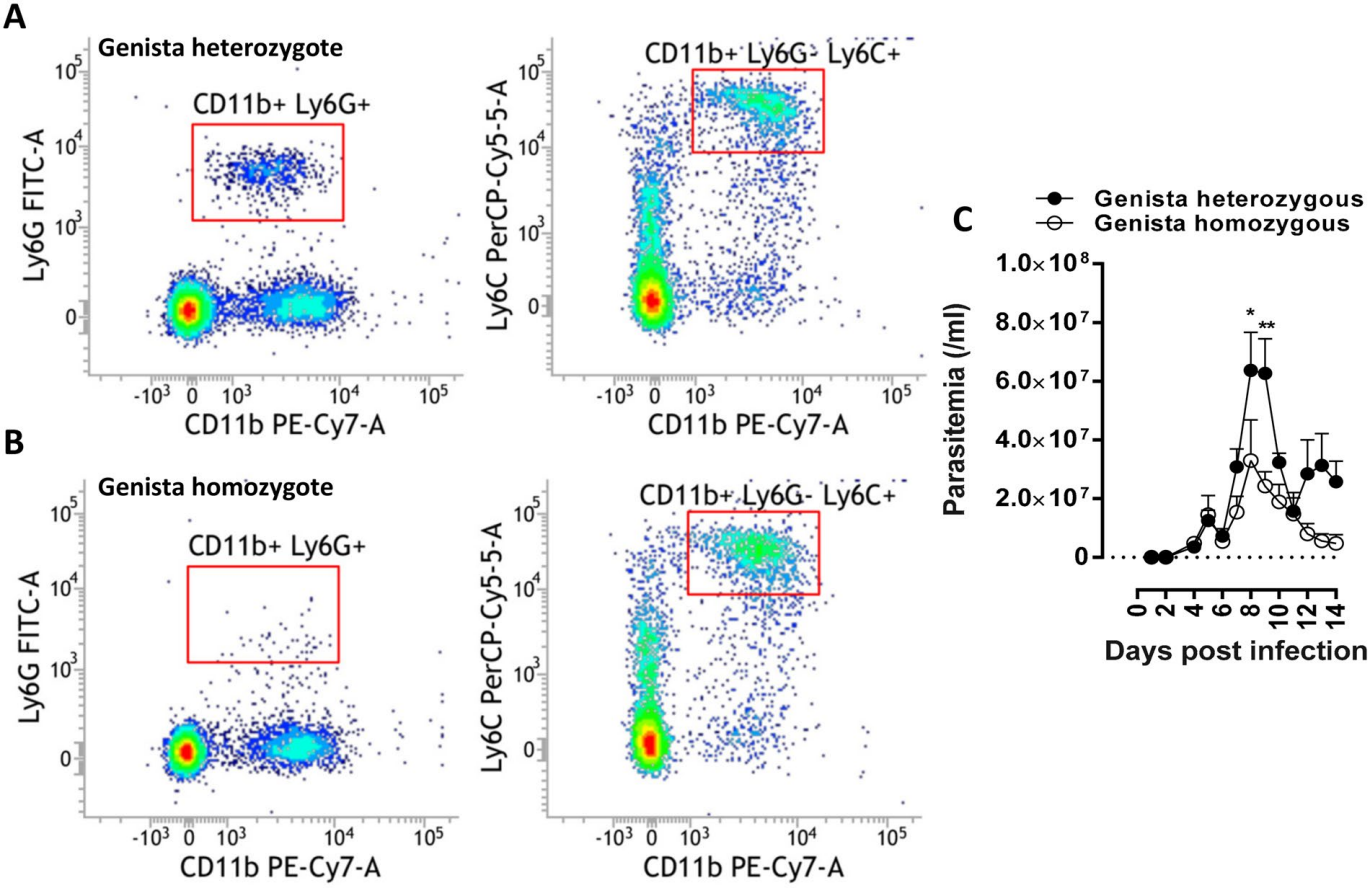
Effect of congenital neutropenia on trypanosome infection progression in *Genista* mice. Flow cytometry analysis on neutrophil and monocyte counts in peripheral blood of (**A**) heterozygous (controls) and (**B**) homozygous *Genista* mice. (**C**) Parasitemia progression in heterozygous (*n* = 6) and homozygous (neutropenic, *n* = 9) *Genista* mice following a tsetse mediated infection with *T.b.b*. AnTAR1. Parasitemia data are the means ± SEM. Data are representative of 2 independent experiments. Statistical significance levels based on the Mann-Whitney test are indicated.

To assess the overall role of neutrophil recruitment during the infection onset (up to 12 dpi), two complementary experimental approaches were used: (*i*) two different transient neutrophil depletion strategies using the rat anti-mouse Ly6G IgG2a (1A8) or the rat anti-mouse Ly6G&Ly6C IgG2b (RB6-8C5) (Fig. 4) and (*ii*) the *Genista* mice^24^ which represent a genetic mouse model of neutropenia (Fig. 5).

**Figure 6:**
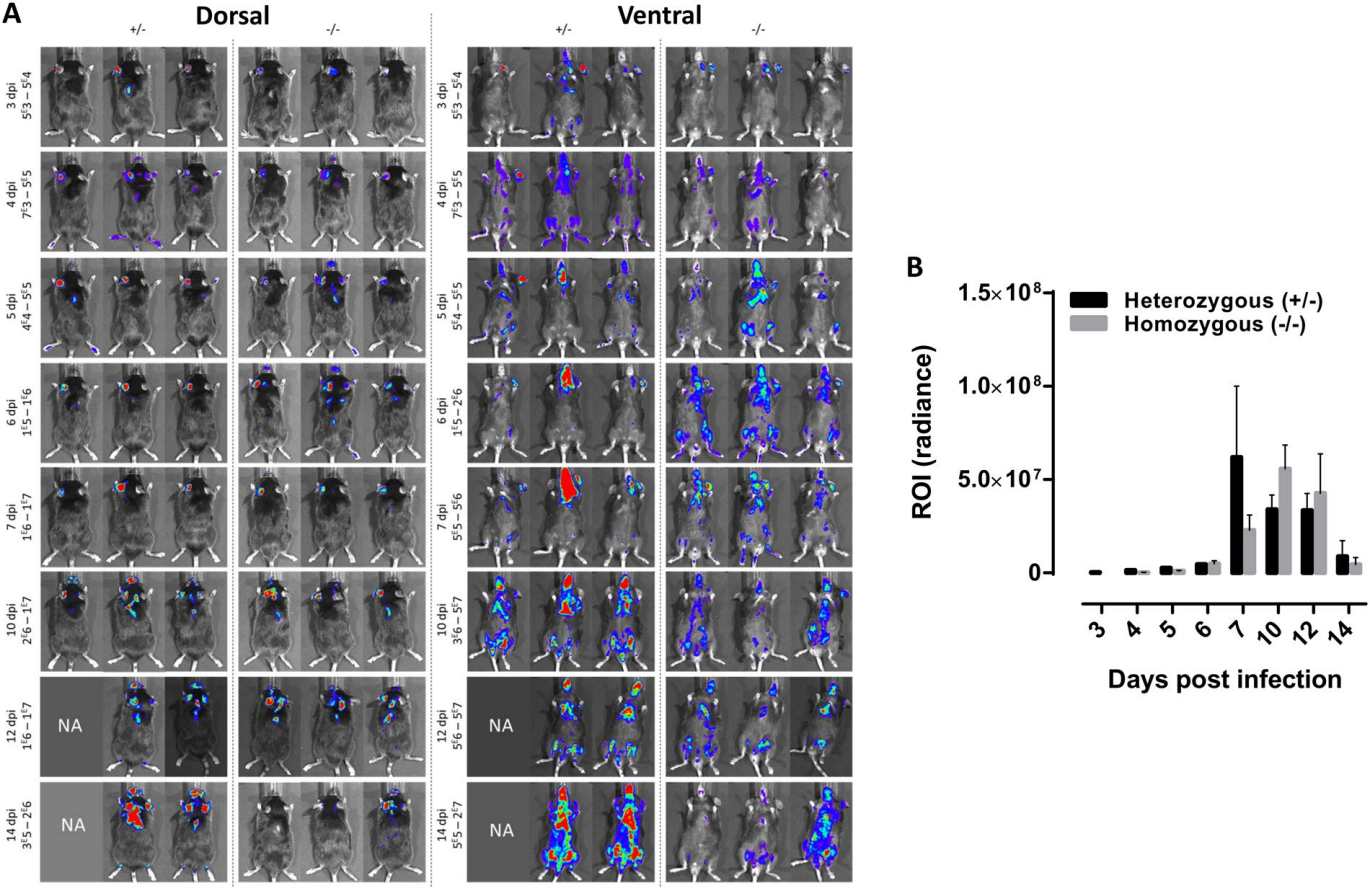
Effect of congenital neutropenia on the dermal trypanosome infection progression in *Genista* mice. BLI analysis of dermal parasite burdens following a *T. brucei* AnTat1.1E^PpyRE9^ infective tsetse fly bite. (**A**) Comparison of the *in vivo* biodistribution and dermal burdens in heterozygous (+/−, *n* = 3) and homozygous (−/−, *n* = 3) *Genista* mice subjected to a dorsal and ventral image analysis. (**B**) Luminescence quantified within a ROI corresponding to the left ear, where parasites were inoculated by a tsetse fly bite. Luminescence was measured using a 3 minute exposure time between 3 and 6 dpi. From 7 dpi onwards, 5 second exposure times were used to avoid saturation. No statistically significant differences were recorded. NA: one animal succumbed within 12 days of infection, precluding image acquisition.

Remarkably, transient depletion of neutrophils (depletion validated in LysM-GFP mice, Fig. S5) following a single injection of a depleting antibody (1A8 versus the appropriate isotype control 2A3) one day prior to infection of C57Bl/6 TNF-α^−/−^ mice (high parasitemia model) using both the 1A8 or the RB6-8C5 depleting antibodies (in comparison with their respective isotype controls 2A3 and LTF-2) resulted in a significant reduction of parasitemia levels in peripheral blood as compared to isotype treated C57Bl/6 TNF-α^−/−^ mice (Fig. 4). Comparing naturally transmitted *T. brucei* infections in homozygous *Genista* mice that are highly neutropenic (Fig. 5A versus 5B) and the heterozygous control littermates unequivocally confirmed elevated trypanosome levels in blood if normal neutrophil levels are present (Fig. 5C). Using BLI, the impact of a neutropenic state on the dermal parasite burdens was evaluated longitudinally (Fig. 6). No significant differences were recorded over the course of infection and maximal dermal burdens were the same in both groups of mice. These data suggest that the presence of recruited neutrophils at the bite site dermal environment do not contribute significantly to parasite elimination and contribute to the development of higher systemic parasitemia levels.

To assess whether neutrophils affect the early inflammatory response against the parasite, plasma cytokine levels were evaluated until day 12 post natural infection, revealing the significant induction of several inflammatory cytokines coinciding with parasite infection, while being absent following a naive tsetse fly bite (Fig. S6A). However, comparison of plasma cytokine responses in *Genista* homozygous and heterozygous mice revealed no significant differences in plasma cytokine responses, except for a higher IL-6 concentration in homozygous mice at 6 dpi (Fig. S6B). Accordingly, in C57Bl/6 TNF-α^−/−^ mice treated with 1A8 and with a significantly lower parasite burden as compared to isotype-treated littermates, no significant changes in the major inflammatory cytokine profile were recorded (Fig. S6B).

## Discussion

In the natural transmission of pathogens by the bite of a blood feeding vector, the host skin forms an important immunological barrier providing the first line of response against the tissue injury and the inoculated pathogen. Here, the main task of this immune response is to contribute to the tissue repair as well as the containment of the invading pathogen to the skin to prevent spreading to inner organs^25^. Neutrophils are prominently and rapidly recruited from the circulation to the bite site of infection. They possess a variety of pathogen-killing mechanisms that can be activated and become effective within hours following infection. However, several pathogens such as the *Leishmania* parasite have evolved different strategies to escape this killing and even enhance the progress of the early infection^9^.

The early immunological reactions following a natural African trypanosome infection initiated by tsetse flies are poorly understood, although they represent a crucial aspect of the parasite transmission cycle and potentially an interphase for intervention. The interaction of trypanosomes with the host immune system is known to be complex and involves the modulation of innate and adaptive responses and an extensive parasite antigenic plasticity^1^. Recently, we have demonstrated that metacyclic trypanosomes that arise in the tsetse salivary glands are highly capable of infecting a mammalian host through the natural intradermal route^3^, despite the fact that the skin represents an important immunological barrier for invading pathogens^15^. Based on infections in C57BL/6 and LysM-GFP mice, neutrophils (CD11b^+^Ly6C^+^Ly6G^+^LysM^−^GFP^Hi^ cells) were found in this study to be the main innate immune cell type recruited rapidly to the dermal site of infection, a feature that was inherent to the biting related tissue damage and irrespective of trypanosome presence. Neutrophil recruitment coincided with transiently upregulated neutrophil chemokines *cxcl1* and *cxcl5* in the dermis. In addition, IL-6, which is a crucial checkpoint regulator for neutrophil trafficking during the inflammatory response by orchestrating chemokine production and leukocyte apoptosis, was found to be strongly upregulated^26^. Neutrophils have previously been found to be present in skin reactions (or chancres) at the site of the infective tsetse fly bite. Parasites were also found to persist and proliferate at the primary site of parasite inoculation where they interacted intricately with adipocytes and collagen and reached parasite levels of >10^5^ in a mouse ear model^3^. This skin-resident parasite population is able to efficiently establish itself, without significant levels of parasite cell death, in the vicinity of neutrophils that accumulated due to a second, parasite infection related neutrophil recruitment wave^3^. This is surprising, given the broad range of potential anti-parasitic effector functions of neutrophils, including phagocytosis, degranulation with the release of microbicidal factors, and NETosis whereby entire nuclear chromatin is expelled into the extracellular environment^10^. We have observed that phagocytosis of parasites by LysM-GFP^+^ neutrophils (and myeloid mononuclear cells) is only apparent upon reduced parasite mobility and cannot be significantly detected during the ascending phase of parasite expansion in the ear dermis. Concomitantly, our *in vivo* BLI analyses revealed that neutrophils do not seem to significantly impact dermal parasite burdens. This is different from the intracellular *Leishmania* parasites that were previously shown to survive in non-phagolytic vacuoles of neutrophils, *i.e*. short-lived cells that are used as a “Trojan horse” to eventually infect macrophages^13,27^. Neutrophils could moreover create an immunologically favorable environment for *Leishmania* by stimulating the recruitment of additional monocytes to the infection site and by the inhibition of the installation of a prominent anti-leishmanial Th1 response^14,28^. It is clear from *Leishmania* research that neutrophils should be considered as important regulators of parasitic infections^11,29^.

Transcription studies on the dermis of infected mice indicated a rapid increase in inflammatory gene transcription during the early hours of infection. This is followed by a fast reduction within 18 h and a second upregulation in infected mice, corresponding with parasite expansion in the ear tissue and a second wave of neutrophil recruitment. The rapid decline of the inflammatory response within 18 hpi could indicate an effect of the tsetse fly salivary components^30,31^ or by *Trypanosoma brucei* proteins, known to limit inflammatory responses^32^. TNF-α also plays an important role in controlling and reducing the parasite burden by acting as a trypanocidal factor^33^ and by stimulating cell recruitment through endothelial cell activation. Neutrophils could constitute a source of TNF-α, but our current and previous transcript studies^30^ did only reveal a mild upregulation of TNF-α expression following early dermal infection. This could result from the inhibition of TNF-α production by the activation of parasite adenylate cyclases, resulting in increased intracellular cAMP levels in phagocytes and the inhibition of TNF-α synthesis^34^. Consistent with these findings, tsetse transmitted ESAG4 dominant negative mutant parasites were shown to be hampered in the early parasitemia onset^19,20^. The prominent upregulation of the type II cytokine IL-10 suggests the potential implication of a secreted trypanosomal protein, *T. brucei* Kinesin Heavy Chain (TbKHC1), which is responsible for the SIGN-R1 receptor-dependent induction of IL-10 production, resulting in arginase-1 activation concomitant with reduction of nitric oxide (NO) synthase activity^32^. This fuels L-ornithine production and the synthesis of polyamines, which are essential nutrients for growth of extracellular trypanosomes in the host. Corroborating that this secreted trypanosome factor is involved in infection onset, tsetse-mediated transmission of TbKHC1 knockout parasites resulted in a reduced parasitemia as compared to wild type parasites^32^.

The role of neutrophils in parasite control has long been understudied due to (i) the limited availability of tools to isolate these cells *ex vivo* devoid of contaminating monocytes, (ii) the inability to culture these cells *in vitro* or (iii) efficiently and specifically deplete these cells *in vivo*. Recently, *Genista* mice were generated, in which neutrophil maturation is hampered due to a recessive mutation in the transcriptional repressor Gfi1^24^. In combination with the conventional depletion strategies using the anti-Ly6G Ab (clone 1A8) or the anti-Ly6G/C Ab (RB6-8C5), we have revealed a parasite-beneficial effect of neutrophils. Indeed, infection experiments in the congenital neutropenic mice and in susceptible mice (TNF-α^−/−^ C57Bl/6 mice) treated with neutrophil depletion antibodies revealed lower parasite levels in the blood circulation associated with the neutropenic state. This suggests that the net neutrophil effect is supporting parasite infection establishment, while some anti-parasitic effector functions might still contribute to infection control.

Collectively, this study unveiled a peculiar outcome of the trypanosome - mammalian host - vector co-evolution whereby parasites escape the killing by the early recruitment of neutrophils into the dermis. Despite the armory of potential anti-pathogen effector functions, the presence of these innate immune cells seems to enhance parasite infection establishment. The underlying molecular and cellular immunological basis of the parasite-beneficial environment created by neutrophils needs to be further explored in future studies.

## Methods

### Ethics statement

Mouse care and experimental procedures were performed under approval of the Animal Ethical Committee of the Institute of Tropical Medicine (Ethical clearance nr. VPU2014-1), the Vrije Universiteit Brussel (Ethical clearance nr. 113-220-1, 15-220-14 and 14-220-31) and the University of Antwerp (Ethical clearance nr. 2017-86). Tsetse fly maintenance and experimental work was approved by the Scientific Institute Public Health department Biosafety and Biotechnology (SBB 219.2007/1410). The experiments, maintenance and care of animals are compliant with Directive 2010/63/EU of the European Parliament and of the Council of 22 September 2010 on the protection of animals used for scientific purposes.

### Animals and parasites

C57BL/6 LysM-GFP and TNF-α^−/−^ mice were available from the Vrije Universiteit Brussel and wild type C57BL/6JRj were obtained from Janvier. In the LysM-GFP transgenic mouse line, eGFP was introduced in the murine lysozyme M locus which results in eGFP expression in neutrophils, monocytes and monocyte-derived dendritic cells. Intense eGFP levels were expressed by neutrophils and monocyte-derived DCs (GFP^Hi^), whereas monocytes (GFP^Int^) show an intermediate eGFP expression level^35^. *Genista* Gfi1^Gen^ mice characterized by the absence of mature neutrophils^24^ were obtained as a kind gift from the Malissen lab. Heterozygous mice that are not hampered by the recessive mutation were differentiated from homozygotes by flow cytometric profiling of the blood and served as littermate controls.

*Trypanosoma brucei brucei* AnTAR1 and three transgenic strains of the pleomorphic *T.b.brucei* AnTat1.1E expressing the red fluorescent protein of a *Discosoma* coral (DsRED, Clontech), a mutant version of the *Aequorea macrodactyla* GFP-like protein (TagGFP2, Evrogen) or a red-shifted firefly luciferase (PpyRE9) were used (kind gift of Dr. Nick Van Reet and Prof. Philippe Büscher). As described elsewhere^3,36^, these parasite strains were used to infect tsetse flies (*Glossina morsitans morsitans*) from the colony at the Institute of Tropical Medicine, Antwerp. Selected salivary gland infected (SG+) flies were used to initiate infections in ketamine (100 mg/kg) and xylazine (20 mg/kg) anesthetized mice by allowing individual flies to bite on the mouse ear dermis. Parasitemia development was monitored by microscopic analysis using improved Neubauer counting chambers and Uriglass disposable slides (Menarini Diagnostics) for parasitema levels <10^7^/mL.

### *In vivo* bioluminescent imaging

Parasite burdens were evaluated by *in vivo* bioluminescent imaging (BLI) following an infective bite with *T. brucei* AnTat1.1E^PpyRE9^ on the left ear. BLI was performed 3 minutes after intraperitoneal (IP) injection of 15 mg/kg D-Luciferin (Beetle Luciferin Potassium Salt, Promega) with the IVIS® Spectrum *In Vivo* Imaging System (Perkin Elmer, Zaventem, Belgium) under 2% isoflurane anesthesia. Luminescence acquisition was performed over a 5 second and 5 minute exposure period from respectively the dorsal and ventral side of the animal. Over a period of 14 days, the evolution of parasite burdens in the ear dermis were assessed as emitted luminescence by image analysis using LivingImage v4.3.1 software within a defined region of interest (ROI).

### Parasite phagocytosis assays

*In vivo T.brucei* AnTat1.1E^dsRED^ parasite phagocytosis was evaluated in the dermis of LysM-GFP mice. Dorsal and ventral ear sheets were separated and mounted with Hoechst 33342 (Molecular Probes). The occurrence of phagocytosis was assessed by confocal microscopy using a Zeiss LSM700 microscope equipped with a ECPlan-Neofluar 40×/1.30 Oil DIC M27 objective lens. Videos of 3 s were obtained by combining 10 consecutive images for the 3 different fluorescent channels in an approximate 8 minute time window. *In vivo* phagocytosis was also evaluated by flow cytometry in diluted blood samples of infected mice (see below). For an *in vitro* phagocytosis assay, we adopted a methodology similar to an erythrophagocytosis assay^37,38^. Briefly, *T. brucei* AnTat1.1E^TagGFP2^ trypanosomes were cultured *in vitro* in HMI-9 medium supplemented with 15% fetal bovine serum (FBS). Parasites were harvested by centrifugation at 870 × *g* and washed twice with PBS. 2 × 10^6^ parasites were labelled for 30 minutes with 120 ng/mL pHrodo red succinimidyl ester (Molecular probes) in a 900 µL volume. The reaction was stopped by the addition of 100 µL FBS and incubation for 10 minutes at ambient temperature. Labelled parasites were subjected to two wash steps with RPMI medium. Parasite viability was very well preserved as confirmed microscopically. 10^5^ pHrodo red labelled AnTat1.1E^TagGFP2^ parasites (50 µL) were incubated with 100 µL 1:100 diluted naive mouse blood in RPMI 1640 without phenol red (Life Technologies™) supplemented with penicillin (10 U/µl), streptomycin (10 U/µl), L-glutamine and 25 U/ml heparin. Parasite phagocytosis was evaluated after a 3 h incubation at 37 °C using flow cytometry.

### Flow cytometry

Blood samples for flow cytometry analysis were obtained from the tail vein and diluted 1:100 in RPMI 1640 without phenol red (Life Technologies™) supplemented with penicillin (10 U/µl), streptomycin (10 U/µl), L-glutamine, 10 µg/ml anti-Fc antibody and 25 U/ml heparin. Dissected ears were separated into ventral and dorsal leaflets and dissociated for 1 hour as described elsewhere^3^ using 100 µg/ml Liberase TL (Roche) and 50 µg/ml DNase (Sigma). Enzymatic dissociation was stopped by addition of ice-cold PBS with 2 mM EDTA and 10% of fetal calf serum. Single cell suspensions were obtained through a 70 µm cell strainer (BD) and collected by centrifugation at 870 × *g* for 8 minutes at 4 °C. 1 µg anti-Fc antibody (2.4G2) was added to each staining reaction as well as 25 U/ml heparin to preserve leucocyte viability^39^. Several staining reagents were used for immune cell identification in the blood samples and blood/trypanosome co-cultures: 7AAD (Via-Probe, BD), anti-CD45 APC-Cy7 (30-F11, ebioscience), anti-CD11b PE-Cy7 (clone M1/70, ebioscience), anti-Ly6C APC (AL-21, AbD Serotec), anti-Ly6G PE or FITC (1A8, Biolegend). The antibodies/staining reagents were used at a 1:600 dilution. At least 2 × 10^5^ events per sample were acquired in a volumetric analysis on the FACSVerse flow cytometer (BD) and analyzed with BD FACSSuite Software. Ear samples were analyzed for the presence of different leukocytes. Debris, dead cells and doublets were gated out by setting a cell gate in the FSC-A/SSC-A density plot, a live cells-gate in the 7-AAD/FSC density plot and a SSC-A/SSC-W singlet selection. On the CD45/SSC density plot, a CD45^+^ gate was set for white blood cells. Within the CD45^+^ gate, CD11b^+^Ly6C^+^Ly6G^+^ (neutrophils) and monocytes (CD11b^+^Ly6C^+^Ly6G^−^) were identified. In LysM-GFP mice, neutrophils were identified as CD11b^+^Ly6G^+^Ly6C^+^GFP^Hi^ and monocytes were identified as CD11b^+^Ly6G^−^Ly6C^+^GFP^Int^.

### RNA extraction and cDNA synthesis

Dissected tissues were treated with RNALater (Qiagen) according to the manufacturer’s instructions and stored at −80 °C. Tissue samples were thawed and weighed and were transferred into lysing matrix D homogenization vessels (MP Biomedicals LLC), containing Lysis/Binding Solution (10–12 µL/mg tissue) (Ambion) followed by homogenization with the FastPrep. Ear samples were homogenized twice at 6.0 m/s for 40 seconds. After homogenization, the lysates were centrifuged at top speed (21130 × *g*) for 2–3 minutes in order to remove tissue debris that may be present in the lysate. RNA isolation was carried out by use of the RNAqueous® Kit (Ambion, life technologies) or the RNeasy Fibrous Tissue Mini Kit (Qiagen) according to the manufacturer’s recommendations. Potential DNA contamination was enzymatically removed with 2U RNAse-free DNase I (Ambion) followed by inactivation using the DNase Inactivation reagent (Ambion) and heat inactivation at 75 °C for 10 minutes. RNA purification with the RNeasy Fibrous Tissue Mini Kit includes an on-column gDNA exclusion step. Concentration and purity of RNA samples was measured by absorbance at 260 nm (A_260_) using a NanoDrop spectrophotometer (Isogen, Life Science ND-1000). Absorbance ratios at 260/280 nm and 260/230 nm were determined as a measure for RNA-purity and integrity. First strand cDNA was synthesized *in vitro* from the purified RNA template with Transcriptor Reverse Transcriptase (Roche) and an oligo(dT)_15_ primer. cDNA samples were stored in aliquots at −20 °C until qPCR analysis.

### Real time quantitative PCR

For a number of the mouse genes related to the innate immune response, validated primers were available from an online repository (http://www.rtprimerdb.org); other primers were designed using Primer Blast, Probefinder (Roche) or Clone Manager professional suite 6.0 and verified for specificity by nucleotide Blast. For primer validation, a 1:5 serial cDNA dilution was tested at a 1:20 dilution in a qPCR reaction using iQTM SYBR^©^ Green Supermix (BioLine) with 500 nM primer concentrations (Table S1). The PCR conditions comprised an initial 10 min polymerase activation at 95 °C followed by 45 cycles, each consisting of a denaturation step at 95 °C for 15 s, 60 s annealing at 60 °C and 60 s elongation at 72 °C. The specificity of the primers was assessed by verifying the presence of a single amplicon by melting curve analysis and gel electrophoresis of the reaction products. As reference genes, *alpha-tubulin 1A* (*tuba1a*), *beta-2 microglobulin* (*b2m*), *eukaryotic translation elongation factor 2* (*eef2*) and *hypoxanthine guanine phosphoribosyl transferase 1* (*hprt1*) were included. Based on the G-norm analysis in the Q base Plus Software (Biogazelle), *tuba1a* and *eef2* were found to be the most suitable reference genes for calculating an integrated normalization factor. After normalization, the relative expression levels were analyzed using GraphPad software 6.0.

### Plasma cytokine analyses

Tail vein blood samples were collected in heparinized capillary tubes and plasma was collected following centrifugation (15 minutes, 10.000 × *g*, 4 °C). Plasma levels of IFN-γ, IL-6, IL-10, IL-12p70, IL1β, TNF-α and CXCL1 were determined using the V-PLEX Custom Mouse Cytokine assay (Meso Scale Discovery, Maryland, USA).

### Graphs and statistical analyses

All graphs were prepared by the use of GraphPad Prism 6.0 software (GraphPad Software). The same software was used for statistical analyses. Expression data were log transformed prior to statistical analyses. Data were represented as means ± standard error of the mean. Statistical comparisons were made using the Mann-Whitney test or the two-way ANOVA with Tukey multiple comparison test. Values of *P* ≤ 0.05 were considered to be statistically significant.

### Data availability

All data generated or analyzed during this study are included in this published article (and its Supplementary Information files).

## Electronic supplementary material

Video S1

Video S2

Supplemental figures
