# Supplementary material for: Neutrophils enhance early Trypanosoma brucei infection onset
Source: Sci Rep. 2018 Jul 25;8:11203. doi: 10.1038/s41598-018-29527-y (PMC6060092; doi:10.1038/s41598-018-29527-y)
Supplement: Supplementary file 3 — Supplemental figures [file 41598_2018_29527_MOESM3_ESM.pdf]

Supplemental Information

Title

Neutrophils enhance early *Trypanosoma brucei* infection onset

Authors and affiliations

Guy Caljon[2*], Dorien Mabille[2], Benoît Stijlemans[3,4], Carl De Trez[4], Massimiliano Mazzone[6,7], Fabienne Tacchini-Cottier[8], Marie Malissen[9], Jo A. Van Ginderachter[3,4], Stefan Magez[4,5], Patrick De Baetselier[3,4] and Jan Van Den Abbeele[1*].

[1]Unit of Veterinary Protozoology, Department of Biomedical Sciences, Institute of Tropical Medicine Antwerp (ITM), Antwerp, Belgium. [2]Laboratory for Microbiology, Parasitology and Hygiene (LMPH), University of Antwerp, Wilrijk, Belgium. [3]Myeloid Cell Immunology Lab, VIB-UGent Center for Inflammation Research, Ghent, Belgium. [4]Unit of Cellular and Molecular Immunology, Vrije Universiteit Brussel (VUB), Brussels, Belgium. [5] Ghent University Global Campus, Incheon, South Korea . [6]Laboratory of Tumor Inflammation and Angiogenesis, Center for Cancer Biology, VIB, Leuven, Belgium; [7]Laboratory of Tumor Inflammation and Angiogenesis, Center for Cancer Biology, Department of Oncology, KU Leuven, Leuven, Belgium [8] Department of Biochemistry, WHO-Immunology Research and Training Center, University of Lausanne, Switzerland, [9] Centre d'Immunologie de Marseille-Luminy, Aix Marseille Université UM2, Inserm U1104, CNRS UMR7280, F-13288, Marseille, France.

Supplemental table

Table S1: List of primers used for the dermal transcriptional analyses. Indicated in the table are the sequences and amplicon sizes of primers designed or retrieved from an online repository (RTprimerDB; indicated with *).

Gene	Discription	Name primer	Seqeunce primer	# nucleotides	Amplicon size (bp)
Il-1b	Interleukin 1 beta	Forward primer*	CAACCAACAAGTGATATTCTCCATG	25	152
		Reverse primer*	GATCCACACTCTCCAGCTGCA	21	
Il-6	Interleukin 6	Forward primer*	CTGCAAGAGACTTCCATCCAGTT	23	70
		Reverse primer*	GAAGTAGGGAAGGCCGTGG	19	
TNF-α	Tumor necrosis factor alpha	Forward primer*	CCAGTGTGGGAAGCTGTCTT	20	101
		Reverse primer*	AAGCAAAAGAGGAGGCAACA	20	
TUBA1A	Tubulin, alpha 1A	Forward primer*	AAGGAGGATGCTGCCAATAA	20	135
		Reverse primer*	GCTGTGGAAAACCAAGAAGC	20	
IL-10	Interleukin 10	Forward primer*	TTTGAATTCCCTGGGTGAGAA	21	68
		Reverse primer*	GGAGAAATCGATGACAGCGC	20	
EEF2	Eukaryotic translation elongation factor 2	Forward primer	CTGTGTCTGTCCAAGTCCCC	20	149
		Reverse primer	TACTTTTCGGCCAGGTAGCG	20	
KC (CXCL1)	Keratinocyte chemoattractant	Forward primer	CCACACTCAAGAATGGTCGC	20	98
		Reverse primer	CCGTTACTTGGGGACACCTT	20	
LIX (CXCL5)	Lipopolysaccharide induced CXC chemokine	Forward primer	CACTCGCAGTGGAAAGAACG	20	100
		Reverse primer	CGTGGGTGGAGAGAATCAGC	20	

Supplemental figures

Figure S1: Gating strategy for dermal neutrophils and monocytes. Flow cytometry-based identification of dermal neutrophils ($CD45^+CD11b^+Ly6C^{Int}Ly6G^+$) and monocytes ($CD45^+CD11b^+Ly6G^-Ly6C^{Hi}$) in the dermis of wildtype C57Bl/6 mice naturally infected with *T.b.b.* AnTat1.1E^{dsRed}.

Figure S2: **Influence of parasite dose on innate immune cell recruitment to the dermal trypanosome infection site.** Recruitment of (**A**) neutrophils ($CD45^+CD11b^+Ly6C^{Int}Ly6G^+$) and (**B**) monocytes ($CD45^+CD11b^+Ly6G^-Ly6C^{Hi}$) to the ear dermis within 4.5 hours after the bites of *T.b.b.* AnTAR1 (high parasite inoculum) or *T.b.b.* AnTat1.1E^{dsRed} salivary gland infected (SG+) tsetse flies (low parasite inoculum). Mice not exposed to tsetse fly bites are included as controls.

Figure S3: *In vivo* trypanosome phagocytosis in murine blood. Flow cytometry analysis of (**A**) *T.b.b.* AnTat1.1E^{dsRed} and (**B**) AnTat1.1$E^{TagGFP2}$ uptake by white blood cells ($CD45^+$ cells) revealing no significant levels of phagocytosis in blood of infected mice.

Figure S4: *In vitro* trypanosome phagocytosis in murine blood. (**A**) Confocal microphotographs and merged image of AnTat1.1$E^{TagGFP2}$ *T.b.b.* stained with the lysosome-stable pHrodo Red (**B**) Flow cytometry analysis to detect phagocytosis of pHrodo Red-stained AnTat1.1$E^{TagGFP2}$ *T.b.b.* parasites by white blood cells ($CD45^+$ cells) in the two fluorescent channels (pHrodo Red and GFP).

Figure S5: **Antibody mediated neutrophil depletion in LysM-GFP mice.** Flow cytometry analysis detecting the presence of neutrophils ($Ly6C^{Int}LysM\text{-}GFP^{Hi}$) and monocytes ($Ly6C^{Hi}LysM\text{-}GFP^{Int}$) in peripheral blood of LysM-GFP mice treated with neutrophil depleting antibody RB6-8C5 (**A**) or 1A8 (**B**) compared to the appropriated isotype controls (LTF-2 or 2A3).

Figure S6: **Plasma cytokine levels following a natural trypanosome transmission.** (**A**) Plasma cytokine levels in mice exposed to naive tsetse flies and in mice at various time points during the early infection onset after a tsetse mediated *T.b.b.* AnTAR1 inoculation. (Control n = 7; naive bite exposed n = 6; infective bite exposed n = 5) (**B**) Plasma cytokine concentrations in naturally 6 day infected *Genista* heterozygous (controls, n = 7) and homozygous (neutropenic, n = 3) mice and in 1A8 treated (neutrophil depleted, n = 6) or 2A3 treated (isotype control, n = 6) C57Bl/6 $TNF^{-/-}$ mice. Cytokine concentrations are the means ± SEM. Statistical significance levels based on the Mann-Whitney test are indicated.

Video S1: Intradermal evaluation of parasite phagocytosis in freshly prepared dermal tissue. Video obtained by confocal imaging (representing approximately 8 minutes of recording) of freshly separated ear dermal sheets of LysM-GFP mice at 90 hpi following the bites of *T.b.b.* AnTat1.1E^{dsRed} infected tsetse flies. Ear tissues were counterstained by mounting in Hoechst 33342.

Video S2: Intradermal evaluation of parasite phagocytosis in conditions of reduced parasite motility. Video obtained by confocal imaging (representing approximately 8 minutes of recording) of separated ear dermal sheets of LysM-GFP mice (harboring $LysM\text{-}GFP^{Hi}$ neutrophils and $LysM\text{-}GFP^{Int}$ myeloid mononuclear cells) at 90 hpi following the bites of *T.b.b.* AnTat1.1E^{dsRed} infected tsetse flies. Ear tissues were counterstained by mounting in Hoechst 33342. Prolonged evaluation of the tissue resulted in reduced motility of the trypanosomes, while myeloid cell patrolling behavior continued and with obvious phagocytic activity towards the dermal trypanosomes.

Figure S1

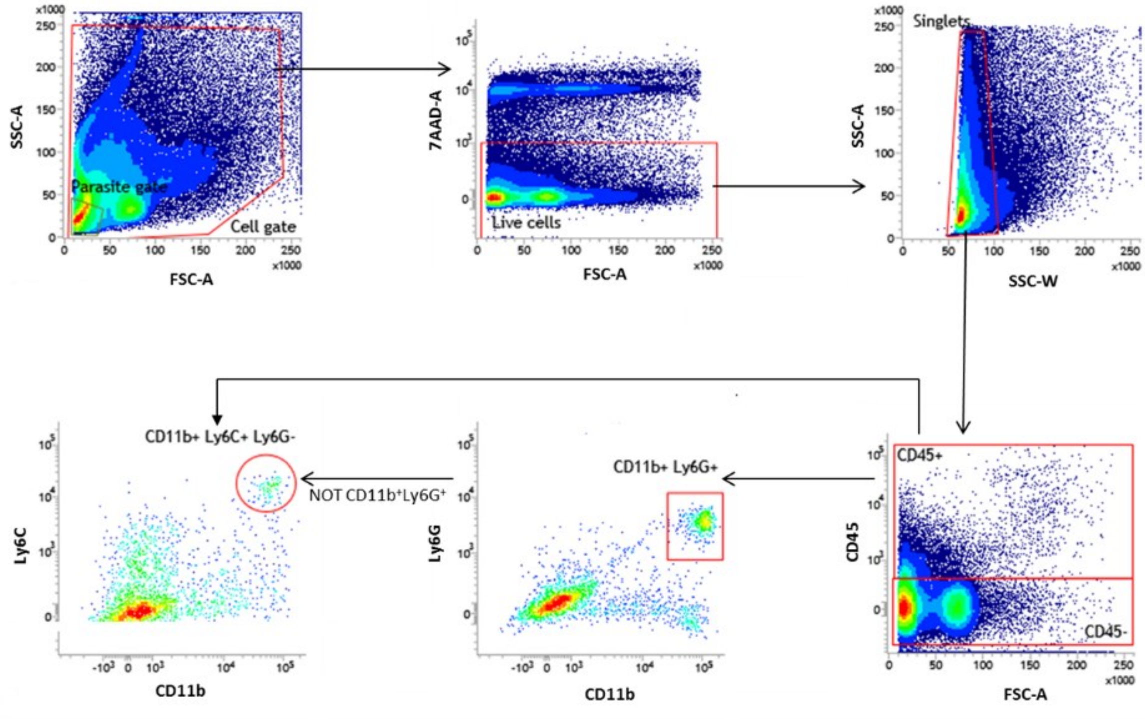

Parasite gate
Cell gate
SSC-A
FSC-A
7AAD-A
Live cells
Singlets
SSC-W
CD45+
CD45-
CD45
CD11b+ Ly6G+
Ly6G
CD11b
NOT CD11b+Ly6G+
CD11b+ Ly6C+ Ly6G-
Ly6C

Figure S2

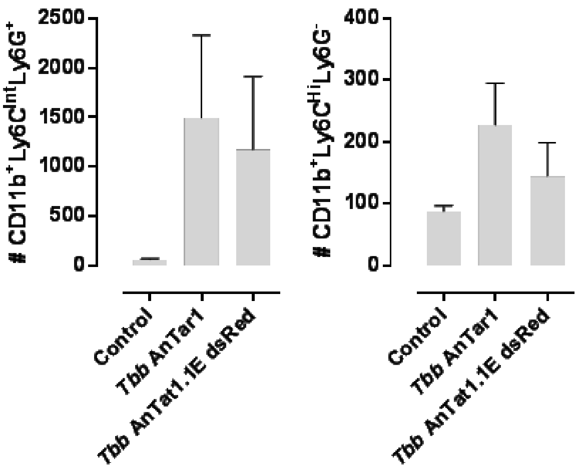

CD11b^{+}Ly6C^{Int}Ly6G^{+}
2500
2000
1500
1000
500
0
Control
Tbb AnTar1
Tbb AnTat1.1E dsRed
CD11b^{+}Ly6C^{Hi}Ly6G^{-}
400
300
200
100
0
Control
Tbb AnTar1
Tbb AnTat1.1E dsRed

Figure S3

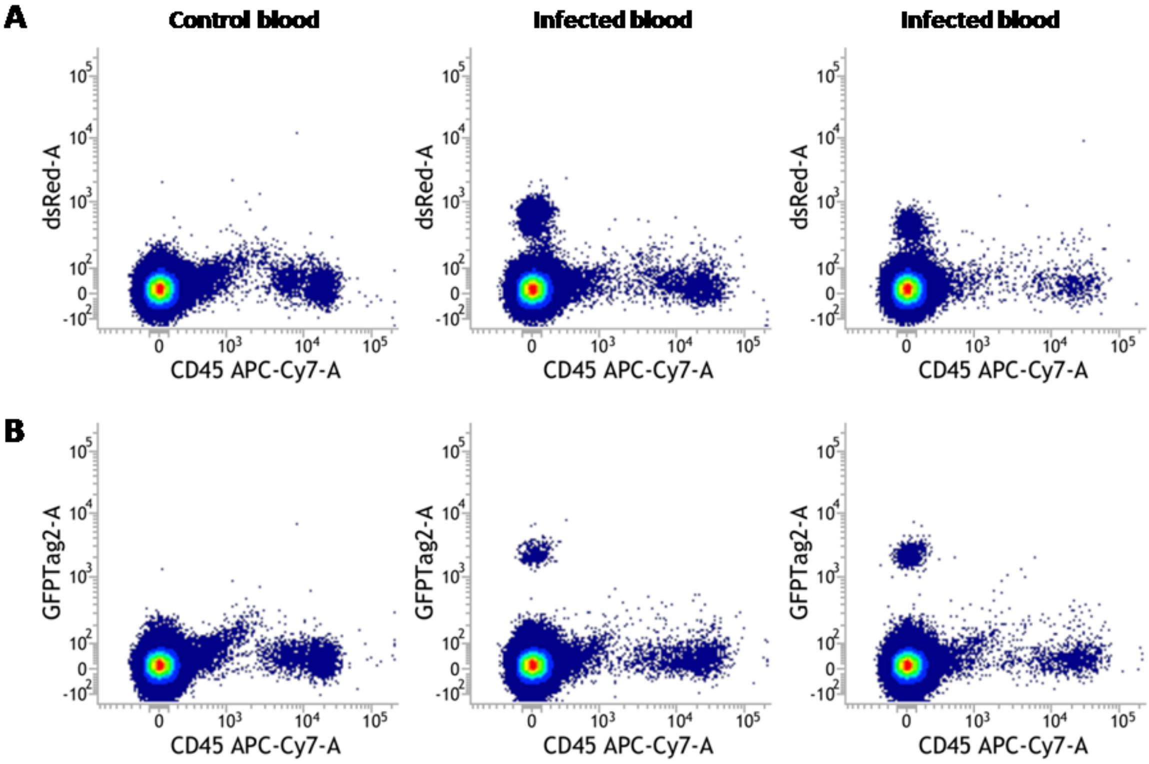
A
Control blood
Infected blood
Infected blood
dsRed-A
CD45 APC-Cy7-A
B
GFPTag2-A
CD45 APC-Cy7-A

Figure S4

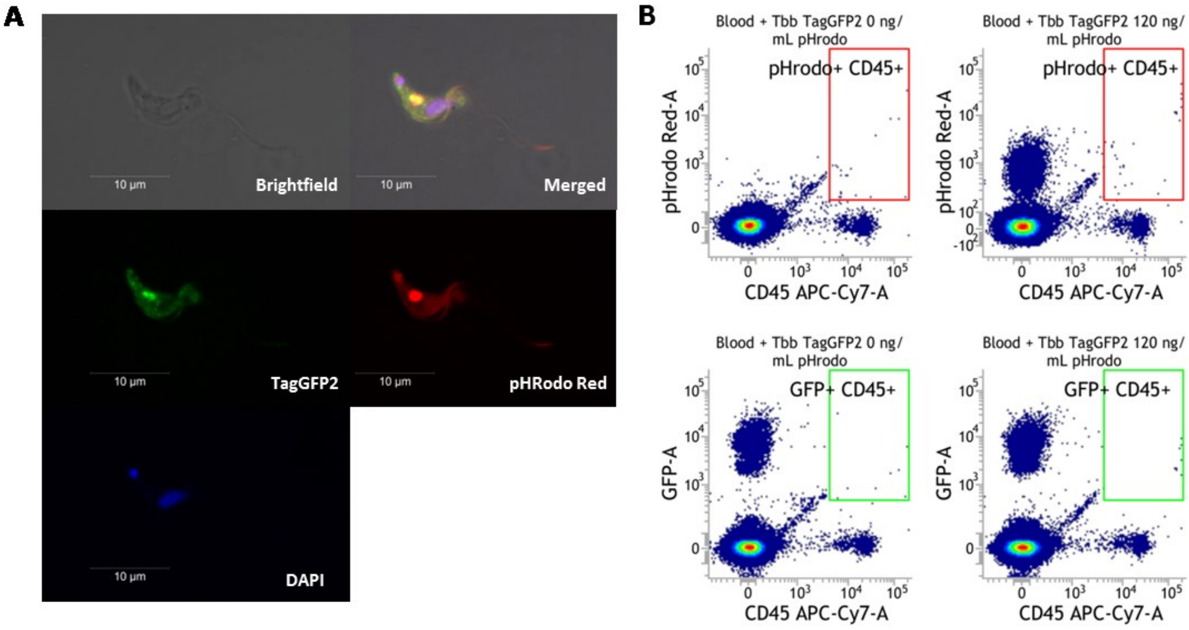
A
10 µm
Brightfield
10 µm
Merged
10 µm
TagGFP2
10 µm
pHRodo Red
10 µm
DAPI
B
Blood + Tbb TagGFP2 0 ng/ mL pHrodo
pHrodo+ CD45+
pHrodo Red-A
CD45 APC-Cy7-A
Blood + Tbb TagGFP2 120 ng/ mL pHrodo
pHrodo+ CD45+
pHrodo Red-A
CD45 APC-Cy7-A
Blood + Tbb TagGFP2 0 ng/ mL pHrodo
GFP+ CD45+
GFP-A
CD45 APC-Cy7-A
Blood + Tbb TagGFP2 120 ng/ mL pHrodo
GFP+ CD45+
GFP-A
CD45 APC-Cy7-A

Figure S5

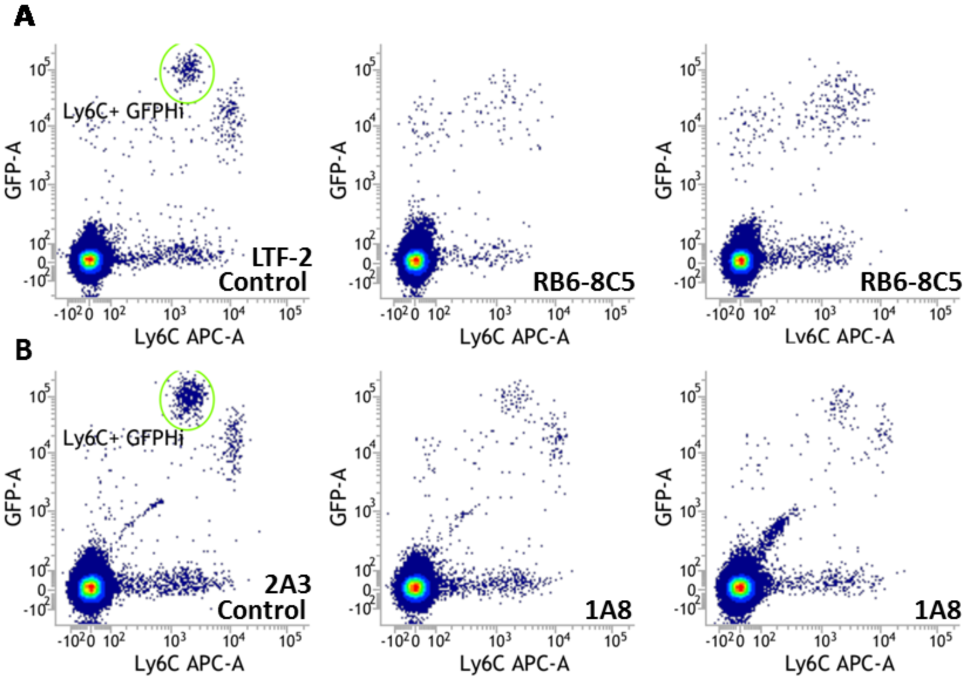
A
B
Ly6C+ GFPHi
LTF-2
Control
RB6-8C5
RB6-8C5
2A3
Control
1A8
1A8
GFP-A
Ly6C APC-A

Figure S6

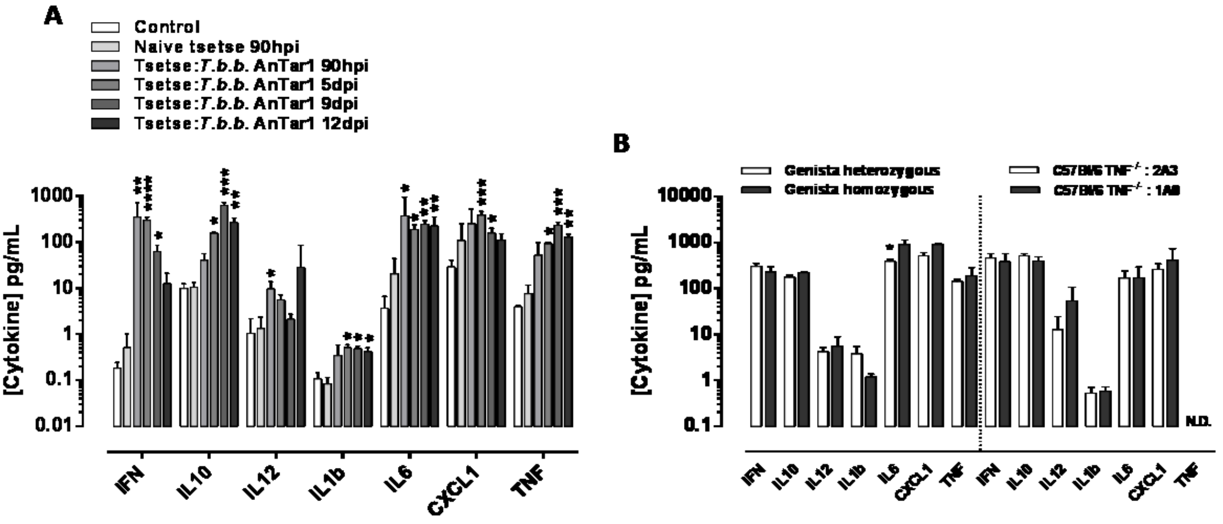

A
Control
Naive tsetse 90hpi
Tsetse:*T.b.b.* AnTar1 90hpi
Tsetse:*T.b.b.* AnTar1 5dpi
Tsetse:*T.b.b.* AnTar1 9dpi
Tsetse:*T.b.b.* AnTar1 12dpi
[Cytokine] pg/mL
1000
100
10
1
0.1
0.01
IFN
IL10
IL12
IL1b
IL6
CXCL1
TNF
B
Genista heterozygous
Genista homozygous
C57Bl/6 TNF-/- : 2A3
C57Bl/6 TNF-/- : 1A8
[Cytokine] pg/mL
10000
1000
100
10
1
0.1
IFN
IL10
IL12
IL1b
IL6
CXCL1
TNF
IFN
IL10
IL12
IL1b
IL6
CXCL1
TNF
N.D.
